# Reassessment of the Role of TSC, mTORC1 and MicroRNAs in Amino Acids-Meditated Translational Control of TOP mRNAs

**DOI:** 10.1371/journal.pone.0109410

**Published:** 2014-10-22

**Authors:** Ilona Patursky-Polischuk, Judith Kasir, Rachel Miloslavski, Zvi Hayouka, Mirit Hausner-Hanochi, Miri Stolovich-Rain, Pinchas Tsukerman, Moshe Biton, Rajini Mudhasani, Stephen N. Jones, Oded Meyuhas

**Affiliations:** 1 Department of Biochemistry and Molecular Biology, The Institute for Medical Research – Israel-Canada, The Hebrew University-Hadassah Medical School, Jerusalem, Israel; 2 Lautenberg Center for General and Tumor Immunology, The Institute for Medical Research – Israel-Canada, The Hebrew University-Hadassah Medical School, Jerusalem, Israel; 3 Department of Cell Biology, University of Massachusetts Medical School, North Worcester, Massachusetts, United States of America; The John Curtin School of Medical Research, Australia

## Abstract

TOP mRNAs encode components of the translational apparatus, and repression of their translation comprises one mechanism, by which cells encountering amino acid deprivation downregulate the biosynthesis of the protein synthesis machinery. This mode of regulation involves TSC as knockout of *TSC1* or *TSC2* rescued TOP mRNAs translation in amino acid-starved cells. The involvement of mTOR in translational control of TOP mRNAs is demonstrated by the ability of constitutively active mTOR to relieve the translational repression of TOP mRNA upon amino acid deprivation. Consistently, knockdown of this kinase as well as its inhibition by pharmacological means blocked amino acid-induced translational activation of these mRNAs. The signaling of amino acids to TOP mRNAs involves RagB, as overexpression of active RagB derepressed the translation of these mRNAs in amino acid-starved cells. Nonetheless, knockdown of raptor or rictor failed to suppress translational activation of TOP mRNAs by amino acids, suggesting that mTORC1 or mTORC2 plays a minor, if any, role in this mode of regulation. Finally, miR10a has previously been suggested to positively regulate the translation of TOP mRNAs. However, we show here that titration of this microRNA failed to downregulate the basal translation efficiency of TOP mRNAs. Moreover, Drosha knockdown or *Dicer* knockout, which carries out the first and second processing steps in microRNAs biosynthesis, respectively, failed to block the translational activation of TOP mRNAs by amino acid or serum stimulation. Evidently, these results are questioning the positive role of microRNAs in this mode of regulation.

## Introduction

TOP mRNAs encode more than ninety proteins of the translational apparatus and are characterized by the presence of an oligopyrimidine tract at their 5′ terminus (5′TOP motif), which comprises their core translation *cis*-regulatory element [1]. The translation of these mRNAs is selectively regulated by mitogenic and oxygen signals that converge at the tuberous sclerosis complex (TSC) dimer, TSC2-TSC21 [2], [3]. The TSC1-TSC2 acts as a GTPase-activating protein (GAP) for Ras-homolog enriched in brain (Rheb), and thereby blocks Rheb activity toward its own target, mammalian target of rapamycin (mTOR) [4], [5]. mTOR operates within two functionally and structurally distinct complexes, mTOR complex 1 (mTORC1) and mTORC2 (reviewed in [6], [7]). The essential core components of mTORC1 are raptor (regulatory-associated protein of mTOR) and mLST8 (mammalian lethal with SEC thirteen 8), whereas, those of mTORC2 are rictor (rapamycin-insensitive companion of mTOR), SIN1 (SAPK-interacting 1) and mLST8.

Rheb-GTP operates as an activator of mTORC1 (reviewed in [8]). Accordingly, deficiency in either TSC1 or TSC2 renders mTORC1 activity completely refractory to mitotic arrest or anoxic signal [3], [9]. Most of the effects of mTORC1 are abolished by rapamycin, an allosteric inhibitor, which exerts its inhibitory effect when complexed with its intracellular receptor, the immunophilin FKBP12 (FK506-binding protein) [10].

Once mTORC1 is activated it regulates protein synthesis by direct phosphorylation of: (a) eukaryotic initiation factor (eIF) 4E-binding proteins (4E-BPs) at multiple sites, which consequently dissociates from and derepresses eIF4E [11]; and (b) ribosomal protein S6 kinase (S6K) at T389 [12], which becomes fully active and affects protein synthesis machinery [1]. A third protein, eukaryotic elongation factor 2 kinase (eEF2K), seems to be indirectly phosphorylated and inactivated by mTORC1, thus leading to dephosphorylation and activation of its substrate eEF2 (reviewed in [13])].

The mTORC2 complex has been implicated in the activation of Akt and protein kinase C [14], [15]. Although only mTORC1 is acutely sensitive to the allosteric inhibitor, rapamycin, newly developed competitive inhibitors that target the catalytic site of mTOR have been shown to potently and directly inhibit both complexes (reviewed in [16]).

Translational control of TOP mRNAs by mitogens or oxygen relies on TSC1, TSC2 and Rheb, as deletion of either of the two TSC proteins or overexpression of Rheb, renders TOP mRNAs refractory to serum deprivation [3], [17], [18]. Accordingly, mTOR is essential for transduction of mitogenic or oxygen signals to translation efficiency of TOP mRNAs, as its knockdown represses the insulin-induced translational activation of TOP mRNAs. However, this mode of regulation appears to rely neither on mTORC1 nor on mTORC2, as deficiency of raptor or rictor exerts marginal or no inhibitory effect, respectively, on the translation efficiency of TOP mRNAs [3], [18].

TOP mRNAs are translationally regulated also by amino acid sufficiency [19], [20]. Amino acid starvation leads to rapid dephosphorylation of S6K1, which can be restored upon readdition of amino acids in an mTORC1-dependent fashion ([6] and references therein). Members of the Rag subfamily of Ras small GTPases (RagA, B, C and D) and the trimeric complex, Ragulator, are essential transducers of amino acids signals to mTORC1 activity [8]. Amino acid stimulation elicits movement of mTORC1 to the lysosomal surface, where Rheb and Ragulator reside. The latter recruits Rag GTPases to the lysosomes in a p62-and vacuolar H^+^-ATPase-dependent manner [21], [22], and thereby participates in mTORC1 activation [9], [23]. In contrast, the pentameric complex, GATOR1, inhibits the mTORC1 pathway by functioning as a GAP for RagA, whereas the trimeric complex, GATOR2 negatively regulates GATOR1 [24].

MicroRNAs (miRs) are short oligonucleotides that function as major regulators of gene expression and function at the posttranscriptional level [25]. It has previously been reported that miR-10a binds the CG-rich sequence immediately downstream of the 5′TOP motif in TOP mRNAs. Furthermore, overexpression of miR-10a selectively enhanced the synthesis of ribosomal proteins in untreated cells and increased the polysomal association of the respective mRNAs in amino-acid-starved cells [26]. These results, therefore, imply that overexpressed miR-10a exerts its positive role in the translational control of TOP mRNAs in a 5′TOP motif-dependent manner.

Here, we set out to establish the pathway that transduce amino acid signal to translation efficiency of TOP mRNAs. Our results show that this pathway relies on TSC and mTOR, exhibits distinct requirement for the small GTPase – RagB. Moreover, amino acid-induced translational activation of these mRNAs, does not depend on either of the two canonical complexes, mTORC1 and mTORC2, or on microRNAs.

## Materials and Methods

Materials used for treating cells, FK506 (20 µM) and puromycin (3 µg/mL) were obtained from Sigma-Aldrich. Rapamycin (20 nM) was from Sigma-Aldrich or Calbiochem. Torin-1 (50 nM) was kindly provided by N. Gray and D. Sabatini, Whitehead Institute for Biomedical Research, Cambridge, MA.

### Cell culture

Human embryonic kidney (HEK) 293 [27], HEK293T cells, as well as MEFs from TSC2^+/+^/p53^−/−^, TSC2^−/−^/p53^−/−^, [28], TSC1^+/+^ and TSC1^−/−^ [29], MDA-MB-231 (human breast adenocarcinoma) [30] and RKO (human colon carcinoma) [31], Dicer^+/+^ and Dicer^−/−^ hemangiosarcoma cells [3] were grown in Dulbecco's modified Eagle's medium (DMEM) containing 10% fetal calf serum, 2 mM glutamine, 100 u/ml penicillin and 0.1 mg/ml streptomycin. Mitotic arrest was achieved by incubation in serum-free medium for 48 h. Amino acid starvation was carried out as previously described [20]. Serum and amino acid starvation was attained by keeping the cells in DMEM (without serum) for 32 h (TSC1 and TSC2) or 18 to 21 h (HEK293) and then for additional 16 h (TSC1 and TSC2) or 3 to 6 h (HEK 293) in Earle's salt solution, MEM-Eagle vitamin solution, 0.37% NaHCO_3_, 100 u/ml penicillin, and 0.1 mg/ml streptomycin. HEK293 and HEK293T cells were transfected using polyethylenimine (PEI) procedure. Briefly, 25 µl of 2 mg/ml PEI (Sigma-Aldrich, average molecular weight 25,000) were added to 750 µl of serum-free medium containing 12.5 µg of DNA. The solution was mixed and kept for 5 min at room temperature prior to its addition to 60 to 70% confluent cell culture in a 100 mm plate containing 10 ml complete medium. The medium was changed the next morning and cells were harvested about 48 h posttransfection.

Polysomal fractionation and RNA analysis were performed as previously described [3]. Typical polysomal profiles of cells with or without amino acids and detailed distribution of rpL32 and actin mRNA along these profiles have been previously shown [20].

### Molecular probes

The isolated fragment probes used in the Northern blot analysis were: a 0.97-kb fragment bearing the *rpL32* processed gene, 4A [32]; a 0.29-kb *Eco*RI-*Hind*III fragment containing mouse *rpS16* cDNA; a 0.85-kb PCR generated fragment containing mouse *rpS6* coding and flanking sequences [33]; a 1.15 kb *Pst*I fragment containing mouse α-actin cDNA [34]; 1.05 *Pst*I fragment containing rat ß-tubulin cDNA [35] and a 0.8-kb *Hin*dIII fragment containing a hGH cDNA.

### Western blot analysis

Immunoblotting was performed as described [36], using antibodies against rpS6 (#2217), phospho rpS6 (Ser235/236 [#2211] or Ser240/244 [#2215]), phospho S6K1(Thr389) [#9206], phospho Akt(ser473) [#4058], P-4E-BP(Thr37/46) [#2855], ß-actin [#4967], α-tubulin [#2144], mTOR [#2972], rictor [#2114] and raptor [#2280] (Cell Signaling Technology, Beverly, MA, USA), as well as FLAG (F3165, Sigma Sigma-Aldrich) and myc (SC-40, Santa Cruz). All antibodies were diluted 1∶1000. Exposures were chosen so that the chemiluminescent signals were within the linear response of the film and were quantified by ImageMaster VDS (Amersham Pharmacia Biotech).

### Knocking down and overexpression using lentiviral vectors

Cloning of an shRNA for the non-relevant HcRed mRNA and knockdown of mTOR and raptor were performed as previously described [18]. The sequences of the HcRed sense and antisense oligonucleotides are:

GATCCCCGTATGCGCATCAAGATGTATTCAAGAGATACATCTTGATGCGCATACTTTTTGGAAA and AGCTTTTCCAAAAAGTATGCGCATCAAGATGTATCTCTTGAATACATCTTGATGCGCATACGGG, respectively. Rictor was knocked down using commercially available lentiviral vector that confer puromycin resistance (TRCN0000074290, Sigma-Aldrich).

FLAG-pLJM1-based plasmids encoding FLAG-RagB and FLAG-RagB (Q99L) were obtained from Addgene (No. 19313 and 19315, respectively) and utilized for generating the respective lentiviruses for infection of HEK293 cells, as described above for shRNA expressing lentiviruses.

The MICB coding sequence was inserted into the SIN18-pRLL-hEFIap EGFP-WRPE instead of the GFP as previously described [37].

Sponges anti-miR-10b and anti–miR-BART 1–5p (control) were excised and cloned into the lentiviral vector SIN18-pRLL-hEFIap EGFP-WRPE, downstream to the GFP cassette. Each sponge consists of 6 adjacent binding sites for the relevant viral miRNA, separated by a 4-nucleotide (AGAG) spacer [38].

### Transfection and Luciferase assay

Dual luciferase PsiCheck2 reporter vectors containing either a fragment from the 3′-UTR of *NCOR2* that bears miR-10a/10b binding site (designated miR-10), or a negative control with a mutated miR-10a/10b seed region (designated miR-10mut) [39] were used. MDA-MB-231 cells expressing anti-miR-10b sponge or a control sponge were grown in RPMI medium and plated in 6-well format, at 6×10^5^ cells/well, just before the transfection took place. Solution of 400 µl serum-free medium, linear PEI and either 6 µg of miR-10 or miR-10mut DNA (in a ratio of 3∶1) were prepared and kept for 15 min. at RT before added to the cells. Medium was changed in the next morning and 4 hours later cells were washed twice with PBS. 150 µl of 1× Pasive Lysis Buffer (Promega) were added to each well and cells were scrapped vigorously with rubber policeman. The lysates were kept on ice for 10 minutes and then frozen at −70°C. Thawed lysates were centrifuged at 9,300 g (4°C) for 10 minutes and aliquots containing 20 µg of protein were used for the assay. Luciferase assay was performed using the Dual-Luciferase Reporter (DLR) Assay System (Promega, USA) and Mithras LB 940 microplate (Berthold Technologies).

### Quantification of MICB and GFP expression

The expression of MICB on the cell surface was quantified by flow cytometry using anti-MICB antibody (MAB1599, R&D Systems), whereas that of GFP by its own fluorescence, using a FACScan (Becton Dickinson Immunocytometry Systems, San Jose, California, USA)

### Quantification of miRs

Total RNA from Dicer^+/+^ and Dicer^−/−^ was prepared using Ultraspec RNA (Biotecx Laboratories, Houston, Texas). miScript Reverse Transcriptase kit and miScript SYBR Green PCR kit (Qiagen, Duesseldorf, Germany) were used for relative quantification of mature miRNA expression levels. Reverse transcription for individual miRNAs or U6 snoRNA were performed according to manufacturer's instructions. Quantitative real-time PCR (qPCR) of the cDNA products were performed using LightCycler 480 Real-Time PCR System (Roche). Analysis was performed using the LightCycler 480 Software. A dilution series using a known cDNA sample was used to generate a standard curve for each assay. The threshold cycle (CT) was determined by automatic assignment of the threshold at the exponential phase of the amplification curves. The following primers were used for qPCR of miR-10a TACCCTGTAGATCCGAATTTGT, miR-140 CAGTGGTTTTACCCTATGGTAG, and U6 GATGACACGCAAATTCGTGAA. Quantitative PCR analysis of mature miRNA in RKO cells as described [40].

## Results

### The translation of TOP mRNAs is resistant to amino acid deprivation in cells deficient of TSC1 or TSC2

**Figure 1:**
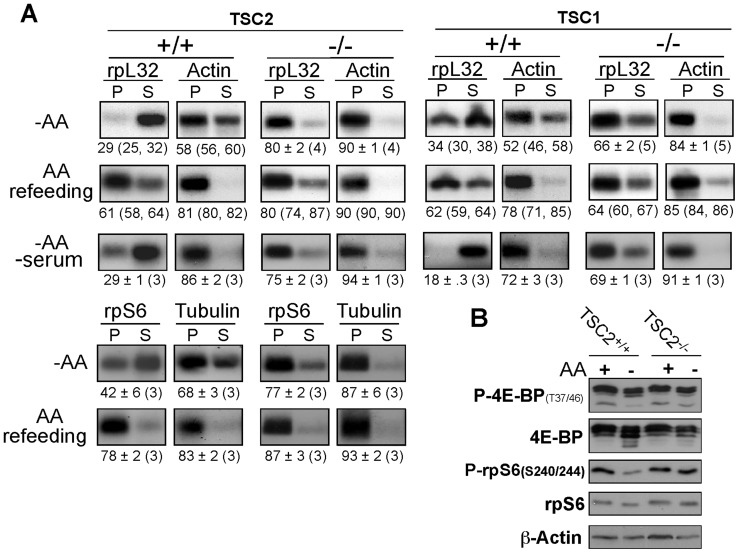
TSC2 or TSC1 deficiency rescues TOP mRNAs from translational repression in amino acid-starved cells. (A) TSC2^+/+^ and TSC2^−/−^ as well as TSC1^+/+^ and TSC1^−/−^ MEFs, were amino acid-starved for 16 h (−AA), amino acid-starved and then refed for 2 h, or amino acid staved during the last 16 h of 48 h serum starvation (−AA −serum). Subsequently cells were harvested and cytoplasmic extracts were prepared. These extracts were centrifuged through sucrose gradients and divided into polysomal (P) and subpolysomal (S) fractions. RNA from equivalent aliquots of these fractions was analyzed by Northern-blot hybridization with cDNAs for rpL32 mRNA (a TOP mRNA) and actin mRNA (a non TOP mRNA) (in the case of TSC2 also with cDNAs corresponding to rpS6 and tubulin). The radioactive signals were quantified, and the relative translational efficiency (% of the P signal relative to the P+S signals) of each mRNA is numerically presented beneath the autoradiograms as percentage of the mRNA engaged in polysomes. These figures are expressed as an average ± SEM of the number of determinations in parenthesis, or the average with the individual values in parenthesis, if only two determinations are presented. (B) TSC2^+/+^, TSC2^−/−^ MEFs were untreated or amino acid-starved for 16 h and then harvested. The cytoplasmic proteins were subjected to Western blot analysis using the indicated antibodies.

The TSC1-TSC2 complex appears to mediate the translational repression of TOP mRNAs upon starvation for serum or oxygen [3], [18]. However, conflicting results regarding the role of this complex in transduction of amino acid signal to mTORC1 activity [41]–[44], rendered questionable its involvement also in transducing the signal of amino acid starvation to translational repression of TOP mRNAs. To directly address this issue, we monitored the translation efficiency of TOP mRNAs, as can be inferred from their relative polysomal association, when TSC2^−/−^ or TSC1^−/−^ MEFs were deprived of amino acids. Fig. 1A demonstrates that the translation of rpS6 mRNA was refractory to amino acid starvation in TSC2^−/−^ MEFs and rpL32 mRNAs in either of these cell lines, in contrast to the apparent sensitivity of these mRNAs in the wild-type counterparts. It is worth noting that the pronounce decrease in the translation efficiency of actin or tubulin mRNA upon amino acid starvation of TSC2^+/+^ and TSC2^−/−^ MEFs (Fig. 1A), relative to that observed in other cell types examined in this study, might simply reflect a cell type-specific response.

Interestingly, the phosphorylation of 4E-BP as well as that of rpS6, was rescued in amino acid-starved TSC2^−/−^ MEFs (Fig. 1B). Previous experiments with the same cell lines have shown an inconsistent sensitivity of the phosphorylation of S6K1 and rpS6 when TSC2^−/−^ MEFs were deprived of both serum and amino acids [42]–[44]. Hence, we set out to examine the translational behavior of rpL32 mRNA in TSC2^−/−^ MEFs that underwent combined starvation. Figs. 1 clearly shows that the deficiency of either TSC1 or TSC2 can rescue the translation efficiency of this mRNA in the absence of both serum and amino acids. These results imply that translational repression of TOP mRNAs by starvation for amino acids, or for amino acids and serum, relies on both TSC1 and TSC2. It is likely therefore, that silencing of the TSC1-TSC2 complex constitutes a critical step toward translational activation of TOP mRNAs by amino acids.

### mTOR is involved in translation control of TOP mRNAs

**Figure 2:**
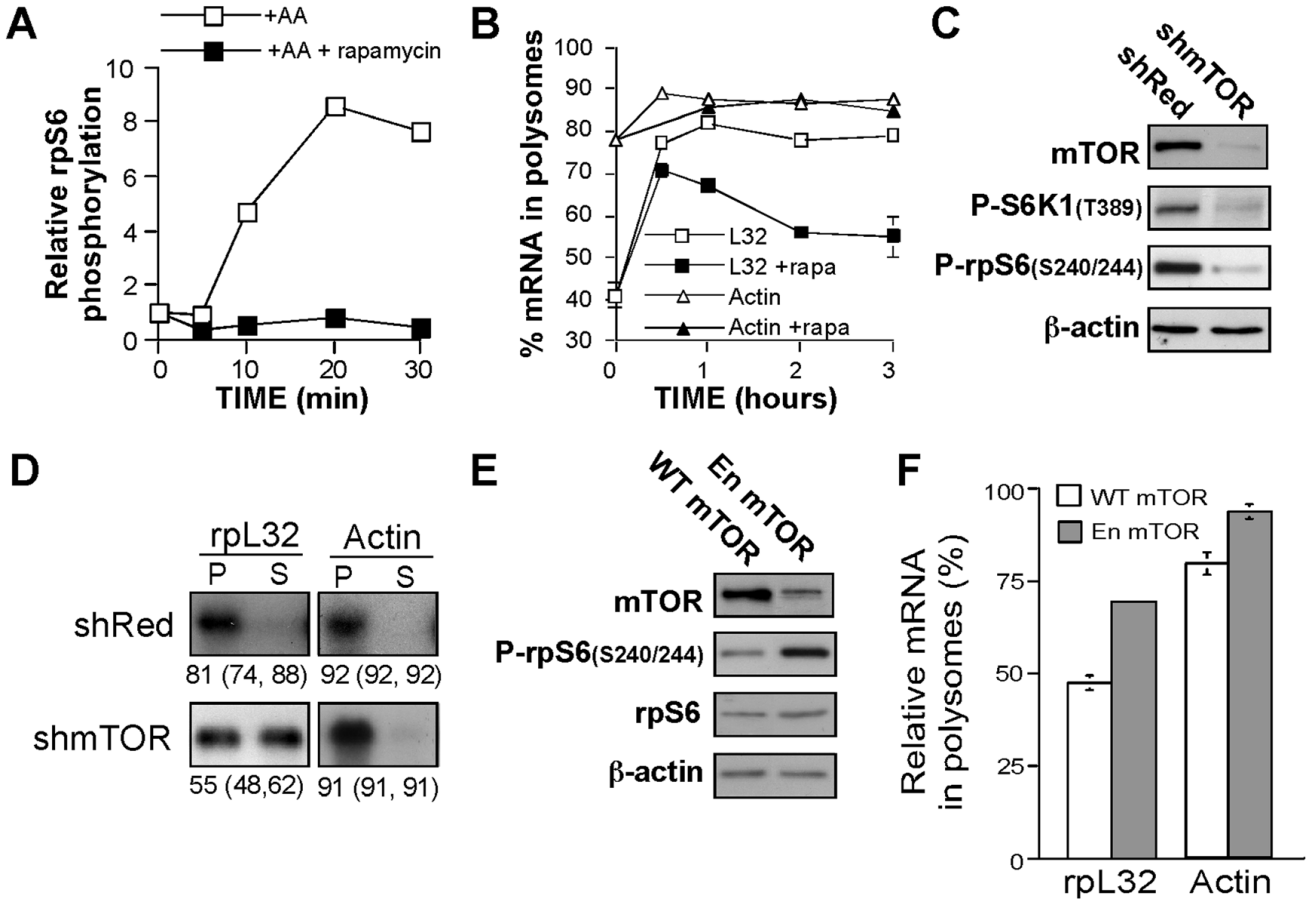
mTOR mediates amino acid-induced translational activation of TOP mRNAs. (A) Kinetics of the effect of rapamycin on mTORC1 activity. 293 cells were amino acid-starved for 2 h and then refed for the indicated time in the presence or absence of 20 nM rapamycin, after which cells were harvested. The cytoplasmic proteins were subjected to Western blot analysis with anti-rpS6 or anti-Phospho-rpS6 antibodies. The chemiluminescent signals of phospho rpS6 were quantified and normalized to those obtained for rpS6 within the same protein extract. The results are numerically presented relative to those obtained for amino acid-starved cells (time zero), which were arbitrarily set at 1. (B) Kinetics of the effect of rapamycin on polysomal association of TOP mRNAs. HEK293 cells were amino acid-starved for 3 h (time zero), and then refed in the absence (open symbols) or presence (filled symbols) of 20 nM rapamycin (rapa). At the indicated times cells were harvested and cytoplasmic extracts were subjected to polysomal analysis. The percentage of mRNA in polysomes at each time point is presented as an average of at least 2 measurements. (C) HEK293 cells were infected with viruses expressing HcRed (Red) shRNA or mTOR shRNA1. Cells were amino acid-starved for 3 h followed by 3 h amino acid stimulation on day 4 post-infection. The abundance of mTOR and its activity were monitored by Western blot analysis of cytoplasmic proteins with the indicated antibodies. (D) Cytoplasmic extracts from cells described in (C) were subjected to polysomal analysis. (E) and (F) HEK293 were transiently transfected with plasmid-based vectors expressing either wild-type (WT) mTOR or enhanced (En) mTOR. 48 h later cells were amino acid-starved for 3 h and harvested. Cytoplasmic proteins were subject to Western blot analysis (E) and cytoplasmic extracts to polysomal analysis (F). The percentage of mRNA in polysomes is presented as an average ± SEM of three experiments.

Rapamycin is a widely used tool for establishing the role of mTOR in many biological processes. However, while this drug inhibited mTORC1 activity with a half-time of about 2 min (Fig. 2A and [45]), it repressed the translation of rpL32 mRNA much more slowly, reaching its maximal effect after 2 h (Fig. 2B). This and previous conflicting reports on the translational repression of TOP mRNAs by rapamycin [2], [18], [20], prompted us to verify the role of mTOR in signaling toward these mRNAs. mTOR knockdown, using lentivirus expressing mTOR shRNA, resulted in downregulation of both mTORC1 activity, as can be judged by the phosphorylation status of S6K and translational activation of rpL32 mRNA upon amino acid stimulation (Figs. 2C and 2D).

The critical role played by mTOR during mouse development has been demonstrated by the death of mTOR-deficient mice shortly after implantation, due to impaired cell proliferation in both embryonic and extraembryonic compartments [46]. It can be argued, therefore, that the repressed translation of TOP mRNAs might reflect a secondary response, due to a mitotic arrest in mTOR knocked down cells. To directly address this issue, we utilized a hyperactive mutant of mTOR (FLAG-mTORS^L1+IT^) that contains four amino acid substitutions within the kinase domain (I2017T, V2198A, L2216H and L2260P), previously shown to rescue mTORC1 activity in amino acid-starved HeLa cells [47]. Indeed, expression of this mutant, but not of wild-type mTOR, alleviated the inhibition of mTORC1 in amino acids-starved HEK 293 cells (Fig. 2E), as well as relieved the translational repression of rpL32 mRNA (Fig. 2F). Taken together, these results imply that mTOR is involved in amino acid signaling to translational efficiency of TOP mRNAs.

### Translational activation of TOP mRNAs by amino acids is resistant to raptor or rictor deficiency

**Figure 3:**
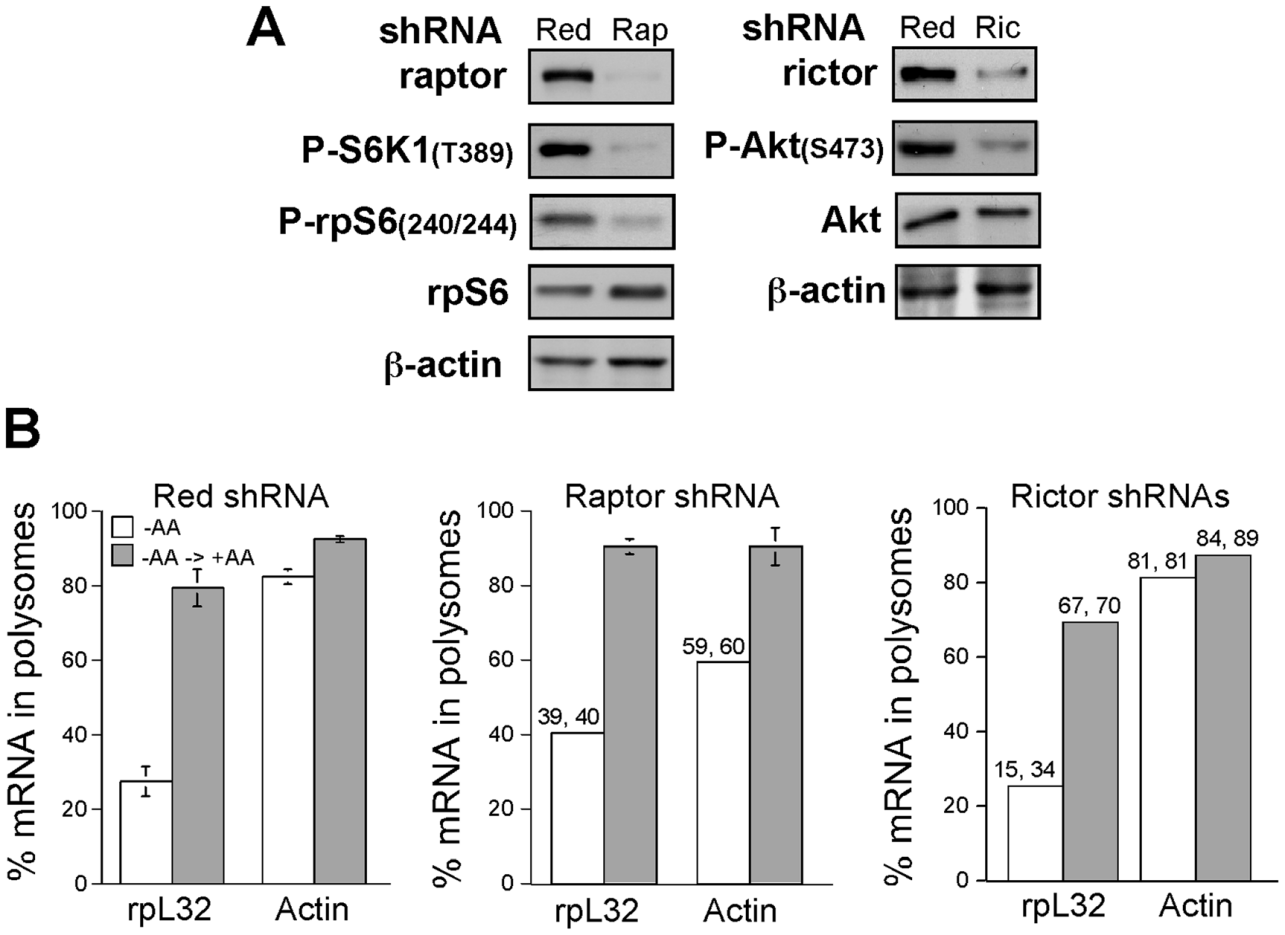
Raptor and rictor are dispensable for translational activation of TOP mRNAs by amino acids. (A) HEK293 cells were infected with viruses expressing Red shRNA, raptor shRNA (Rap) or rictor shRNAs (Ric). The abundance of raptor or rictor, as well the phosphorylation status of direct and indirect substrates of the respective complexes, mTORC1 and mTORC2 (left and right, respectively), was monitored by Western blot analysis. (B) HEK293 cells infected with viruses expressing HcRed, raptor or rictor shRNA were amino acid starved for 3 h (−AA) or starved and then refed for 3 h (−AA→+AA). Cytoplasmic extracts from these cells were subjected to polysomal analysis and the data are presented as described in the legend to Fig. 2F. Numbers above bars are individual values, when only two measurements were performed.

Establishing the role of mTOR in amino acid-mediated translational activation of TOP mRNAs has further underscored the discrepancy between the promptness and sensitivity of mTORC1 response to rapamycin and the relative rapamycin resistance exhibited by TOP mRNAs (Fig. 2 and [20]). To examine whether mTORC1 is involved at all in amino acid-induced translational activation of these mRNAs, we knocked down its core constituent, raptor, in HEK293 cells. This silencing, indeed, led to a pronounced decrease in raptor level and mTORC1 activity, as exemplified by the hypophosphorylation of S6K1 and rpS6 (Figure 3A). However, despite this efficient elimination of raptor, the translational activation of rpL32 mRNA by amino acids was unaffected (Fig. 3B).

The lack of an effect of raptor knockdown prompted us to examine the role ofmTORC2 in this mode of regulation. The results presented in Figure 3A and 3B clearly show that amino acid-induced translational activation of rpL32 mRNA was completely refractory to the loss of rictor, and consequently to that of mTORC2 activity (as can be judged by the hypophosphorylation of Ser473 in Akt in rictor knocked down cells). Notably, we have previously utilized inducible raptor and rictor cell lines (iRapKO and iRicKO, respectively) to demonstrate the dispensability of mTORC1 or mTORC2 in insulin or oxygen induced mTOR translational activation of TOP mRNAs [3], [18]. However, these cell lines exhibit an inherent resistance to prolonged amino acid starvation (data not shown), and therefore could not be used here. Nevertheless, collectively our results imply that amino acid-stimulated translational activation of TOP mRNAs does not rely on raptor or rictor.

### mTOR regulates TOP mRNA translation through its kinase activity

**Figure 4:**
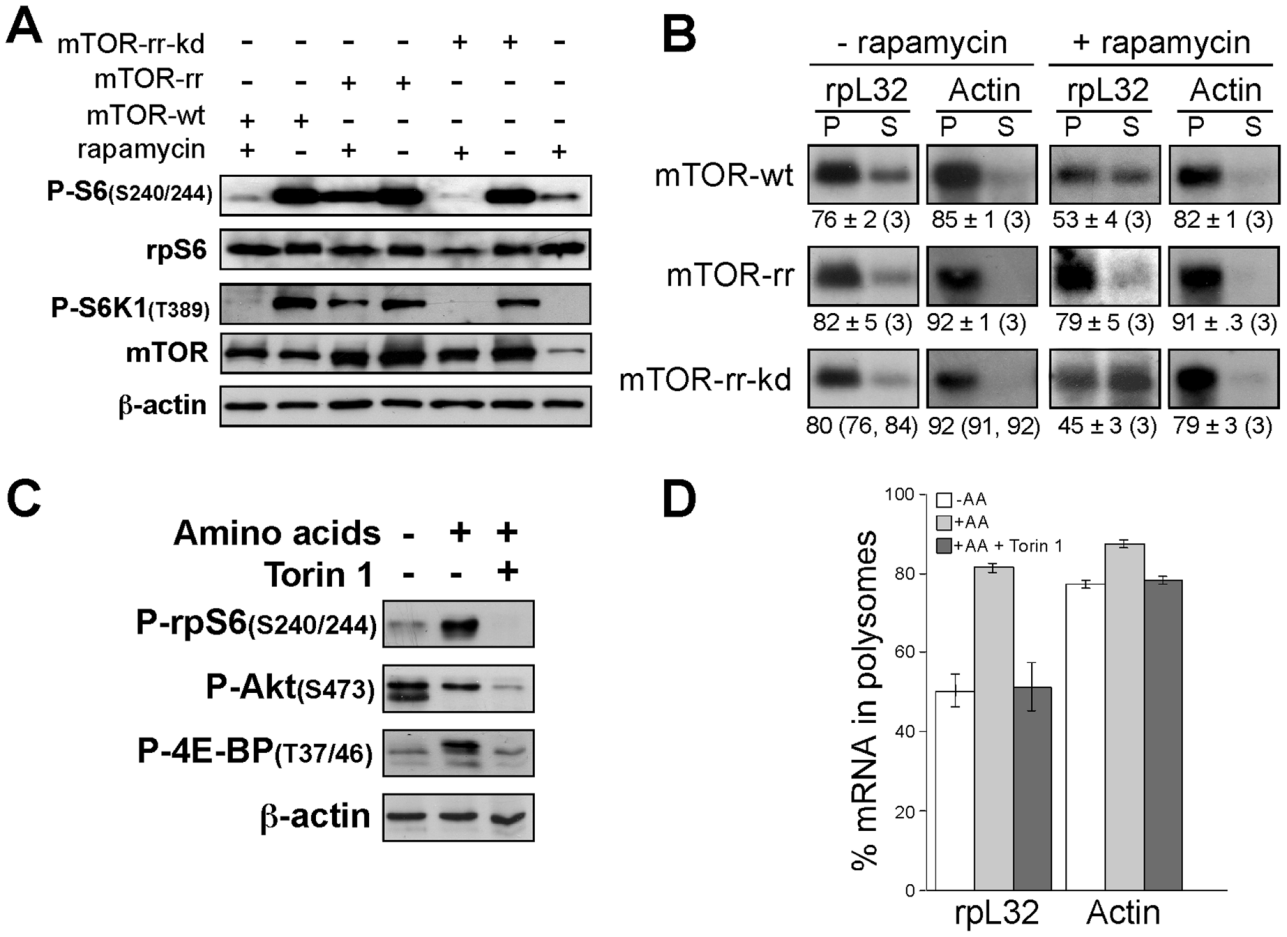
The kinase activity of mTOR is essential for translational control of TOP mRNAs. (A) HEK293 cells were transfected with vectors expressing mTOR-wt, mTOR-rr or mTOR-rr-kd, two days later the cells were amino acid-starved for 3 h followed by 3 h refeeding without or with 20 nM rapamycin. Cytoplasmic proteins derived from the cells were subjected to Western blot analysis with the indicated antibodies. B) Cytoplasmic extracts derived from cells treated as described in (A), were subjected to polysomal analysis. C) HEK293 cells were amino acid-starved for 3 h, or amino acid-starved for 3 h followed by 3 h refeeding without or with 50 nM Torin1. Cytoplasmic proteins derived from the cells were subjected to Western blot analysis with the indicated antibodies. D) Cytoplasmic extracts derived from cells treated as described in (C) were subjected to polysomal analysis and the percentage of mRNA in polysomes is presented as an average ± SEM of three experiments.

One plausible explanation for the apparent ability of mTOR to regulate TOP mRNA translation in an either mTORC1- or mTORC2-independent fashion might be its role as a scaffold protein, rather than an active kinase. Indeed, such a role has previously been proposed for regulation of dystrophin gene expression by mTOR [48]. Two complementary experimental approaches were used to directly address this possibility: a) HEK293 cells were transfected with wild type mTOR [49], rapamycin-resistant mTOR mutant (mTOR-rr) that contains S2035I substitution, or catalytically inactive version of mTOR-rr that contains an additional D2338A substitution (mTOR-rr-kd) [50]. Cells were amino acid-starved and then were refed in the absence or presence of rapamycin. The results show that overexpression of mTOR-rr, but not mTOR-rr-kd or wild type mTOR, can rescue both mTORC1 activity (Fig. 4A) and the translation efficiency of TOP mRNAs (Fig. 4B). b) HEK293 cells were amino acid-starved and then were refed with or without Torin1, a selective ATP-competitive mTOR inhibitor that can block all examined mTOR activities [3], [51]. Indeed, this inhibitor fully suppresses the amino acid-induced translational activation of rpL32 mRNA, to the same degree (about 50% in polysomes) as do rapamycin and mTOR knockdown (compare Fig. 4D with Figs. 2B and 2D). Taken together, these results clearly attest to the positive regulatory role of mTOR catalytic activity in amino acid-induced translational activation of TOP mRNAs.

**Figure 5:**
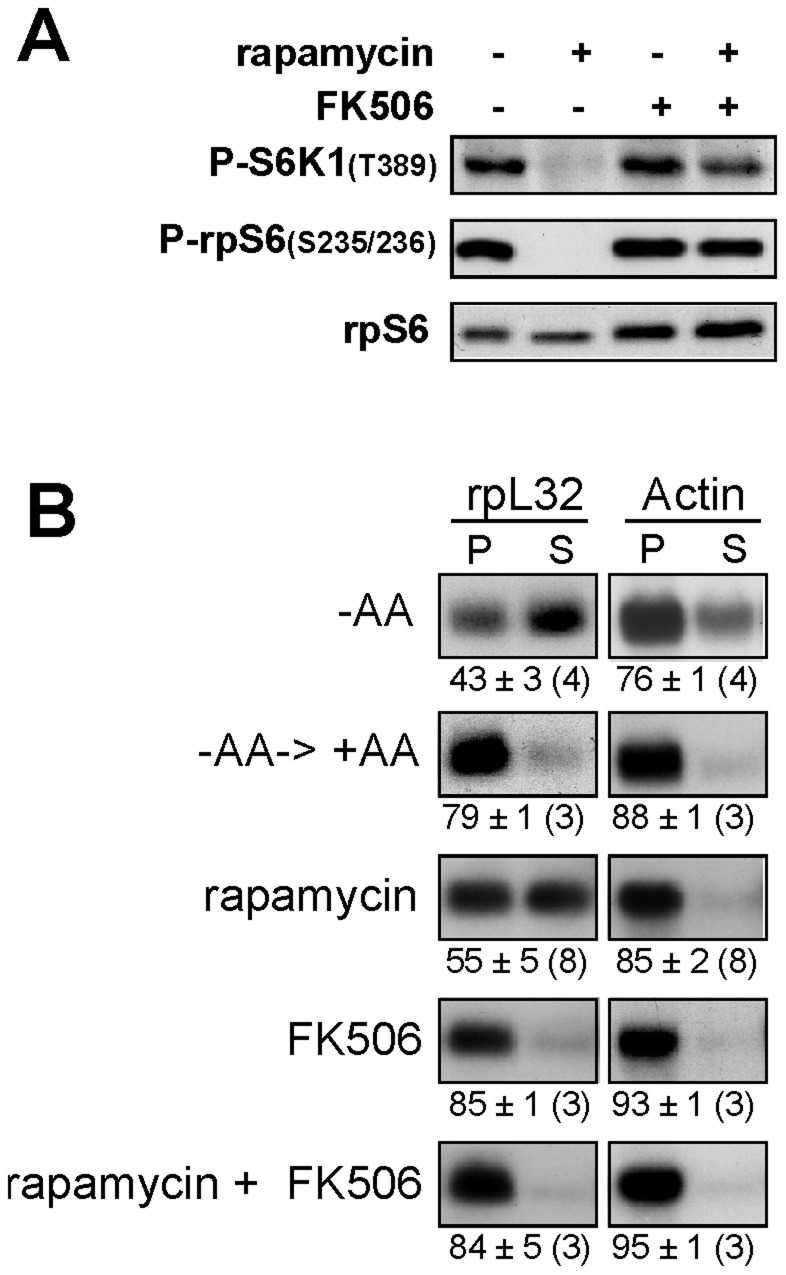
Rapamycin represses the translation of TOP mRNAs in an FKBP-12-dependent fashion. (A) HEK293 cells were amino acid-starved for 3 h and then refed for 3 h in the absence or presence of rapamycin (20 nM), FK506 (20 mM), or both. Cytoplasmic proteins were subjected to Western blot analysis. (B) HEK293 cells were amino acid-starved for 3 h (−AA), refed for 3 h (−AA→+AA) in the absence or presence of rapamycin (20 nM), FK506 (20 mM) or both. Cytoplasmic extracts were subjected to polysomal analysis.

The relative resistance of amino acid-induced TOP mRNAs translation to raptor or rictor knockdown on the one hand (Fig. 3), and the apparent requirement for mTOR activity on the other (Fig. 4), posed a question whether FKBP12, which is known to mediate the inhibition of mTOR by rapamycin, is also involved in the translational repression of these mRNAs. To this end, we utilized FK506, a small molecule that competes with rapamycin for binding to FKBP12. Indeed FK506 can relieve mTOR inhibition, as monitored by phosphorylation of S6K1 and rpS6 (Fig. 5A), as well as the translational repression of TOP mRNAs (Fig. 5B).

Collectively, our results indicate that rapamycin suppresses the amino acid-induced translational activation of TOP mRNAs by the availability of FKBP12, as it does for mTORC1 and mTORC2 activity [52], even though both raptor and rictor seems dispensable for the translational activation.

### Over expression of constitutively active RagB can derepress the translation of TOP mRNAs in amino acid-starved, but not in oxygen-deprived cells

**Figure 6:**
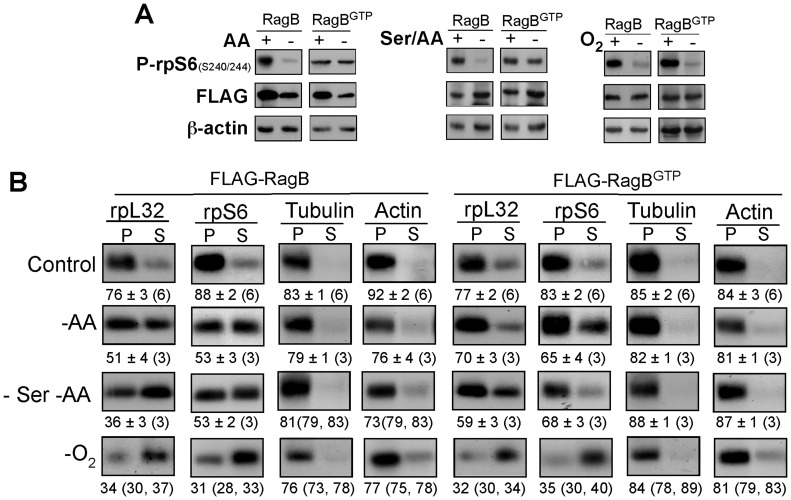
Overexpression of RagB^GTP^ can derepress TOP mRNA translation in amino acid-starved cells, but not in cells deprived of oxygen. (A) HEK293 cells were infected with lentiviral expression vectors encoding FLAG-RagB or FLAG-RagB^GTP^. 48 h post infection cells were subjected to selection by puromycin and 48 h later were either kept untreated (+), amino acid-starved for 8 h (−AA), amino acid starved during the last 3 h of 24 h serum starvation (–Ser/AA) or deprived of oxygen (−O_2_) for 16 h. Cells were harvested and their cytoplasmic proteins were subjected to Western blot analysis using the indicated antibodies. (B) Polysomal analysis of cytoplasmic extracts from cells treated as describe in (A).

Rag GTPases bind raptor and thus mediate amino acid signaling to mTORC1 [9]. Accordingly, expression of constitutively active mutant forms of RagA or B (GTP-bound) can protect mTORC1 activity in amino acid-deprived cells [9], [53]. However, the establishment of raptor as dispensable for amino acid- or oxygen-mediated translational activation of TOP mRNAs (Fig. 3B and [3]), raised a question regarding the role of Rag in this mode of regulation. To examine this issue, we measured the effect of FLAG-tagged wild type RagB or a mutant [RagB (Q99L)] that constitutively binds GTP (RagB^GTP^), on the translation efficiency of TOP mRNAs under stress conditions. Our results show that RagB^GTP^, but not wild type RagB, exerts complete, partial or no protective effect on rpS6 phosphorylation in cells that were amino acid-starved, starved for both serum and amino acids, or oxygen-deprived cells, respectively (Figs. 6A). Notably, RagB^GTP^ exerts a similar stress-specific relief of the translational repression of rpL32 and rpS6 mRNAs (Fig. 6B), suggesting that RagB primarily mediates signals emanating from amino acids.

### TOP mRNAs are not positively regulated by microRNAs

The demonstration that overexpression of miR-10a increases the polysomal association of TOP mRNAs in amino acid-starved cells has implied that this miR positively regulates the translation of TOP mRNAs [26]. However, based on multiple examples of erroneous conclusions regarding the function of an overexpressed protein [19], we set out to examine the role of miRs in this mode of regulation by a loss-of-function approach. Notably, miR-10a and miR-10b differ by just one nucleotide at a non-seed sequence, yet display a similar enhancing effect on the translation of a reporter TOP mRNA [26]. Likewise, miR-10a and miR-10b are potent inducers of neuroblastoma cell differentiation through targeting of nuclear receptor corepressor 2 (NCOR2) [39]. Hence, we initially used the miRNA sponge technique that can block the activity of miR-10b and conceivably that of miR-10a as well [54].

**Figure 7:**
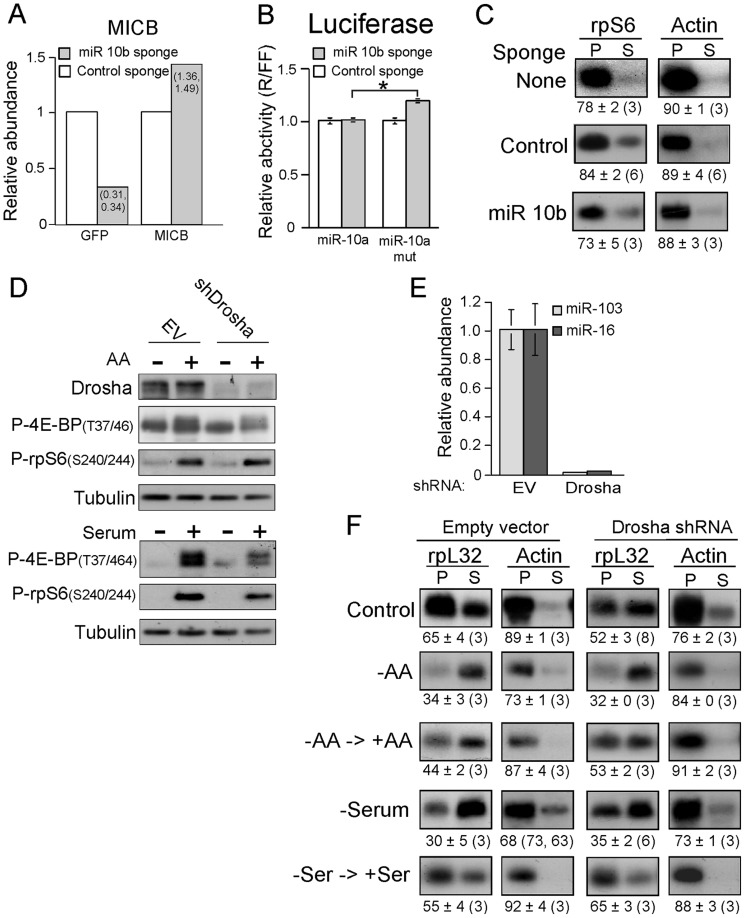
Knockdown of miRs fails to suppress translational activation of TOP mRNAs. (A) MDA-MB-231 cells were infected with lentivirus expressing either anti-miR 10b sponge or anti-miR-BART 1–5p sponge (control). The fluorescent signals of MICB and GFP were analyzed by FACS. The mean intensity of MICB or GFP in the control miR–transduced cells was arbitrarily set up to be 1, and the relative increase in the MICB expression or the decrease in the GFP fluorescence in sponge-10b–transduced cells was calculated accordingly (individual numbers are presented within the bars). (B) MDA-MB-231 cells infected with lentiviruses described in (A) were transiently transfected with Dual luciferase PsiCheck2 reporter vectors. These vectors contained within the 3′ UTR of the Renilla luciferase either a fragment from the 3′-UTR of *NCOR2* that bears miR-10a/10b binding site (designated miR-10), or a negative control with a mutated miR-10a/10b seed region (designated miR-10mut). The Renilla to Firefly activity ratio (R/FF) was calculated for each sample and the average obtained for the miR-10b sponge-infected cells was normalized to that obtained for the control sponge-infected cells, which arbitrarily was set at 1.0. (*) *p*<0.001 versus miR-10a transfected cells (*n* = 8). (C) MDA-MB-231 cells that were either kept uninfected (None), expressed anti-miR 10b sponge or control sponge (Control) were kept untreated and cytoplasmic extracts from these cells were subjected to polysomal analysis. (D) and (F) RKO cells infected with Sin 18, an empty lentiviral vector (EV), or by lentivirus expressing shDrosha RNA. Cells were either untreated [Control in (F)], starved for serum for 19 h and during the last 3 h also for amino acids and then either kept without serum and amino acids [− in (D); −AA in (F)] or refed for just amino acid for additional 2 h [+ in (D); −AA→+AA in (F)]. Similarly infected cells were serum starved for 48 h [−in (D); –Serum in (F)] or serum starved for 48 h and then serum refed for 3 h [+ in (D); –Ser →+Ser in (F)]. Cells were harvested and subjected to Western blot analysis with the indicated antibodies (D) or subjected to polysomal analysis (F). (E) Total RNA was prepared from RKO cells infected with either empty lentiviral vector (EV) or lentivirus expressing shDrosha RNA. The abundance of each of the indicated miRs in Drosha knockdown cells was normalized to that in cells infected with empty vector, which was arbitrarily set at one.

The location of the sponge sequence downstream of the GFP open reading frame enabled us to assess the sponge activity. Thus, the sequestration of the relevant miRNA in MDA-MB-231 cells indeed led to reduced GFP fluorescence intensity (Fig. 7A). Moreover, miR-10b has been implicated in downregulation of the stress-induced cell surface molecule, MICB (MHC class I chain related gene B) [38], and therefore, titrating out this miR resulted in increased expression of MICB (Fig. 7A). Next, we set to examine the ability of miR-10b sponge to derepress the expression of a luciferase encoded by an mRNA that contained in its 3′ untranslated region (3′ UTR) the wild-484 bp-long 3′ UTR from NCOR2 mRNA (designated as miR-10a) [39]. To this end, MDA-MB-231 cells were transiently transfected with miR-10a luciferase reporter. This reporter construct showed a small (about 19%), yet statistically significant, increase in luciferase activity expressing mir-10b sponge, but not a control sponge. Moreover, a reporter containing NCOR2 3′ UTR with mutated miR-10a binding motif (designated miR-10a mut), failed to respond to the expression of the miR-10b sponge (Fig. 7B).

Collectively, these results indicate that miR-10b sponge can titrate out both miR-10a and miR-10b, and thereby set the stage for verifying the role of these miRs in controlling the translation efficiency of TOP mRNAs. Fig. 7C, clearly demonstrates that expression of a mir-10b sponge exerted no effect on the translation efficiency of rpS6 mRNA, when compared to untreated cells or cells transfected with a control sponge. It appears therefore, that miR-10a or miR-10b are not critical for efficient basal translation of TOP mRNAs. It can be argued that the positive effect of these miRs can only be detected under stress conditions. Hence, we set out to examine the effect of global deficiency of miRs on translation efficiency of TOP mRNAs under such conditions.

First, we knocked down Drosha, the nuclear RNase III enzyme that initiates the processing of miRs. The efficient silencing of Drosha (Fig. 7D) indeed, nearly nullified the abundance of representative miRs (Fig. 7E). This global deficiency in miRs had no appreciable effect on the proliferation rate of the respective cells (data not shown), yet it slightly decreased the basal translational efficiency of rpL32 mRNA (see Control in Fig. 7F). Nonetheless, it failed to block the translational activation of this mRNA in response to refeeding of starved cell with either amino acids or serum (Fig. 7F). These results imply that miRs are not required for the recovery of TOP mRNA translation from the nutritional stress. Unexpectedly, however, cells infected with the empty vector were the ones that exhibited minor or incomplete recovery of the translation efficiency of rpL32 mRNA following amino acid or serum refeeding, respectively. The reason for this effect of the empty virus is currently unclear.

**Figure 8:**
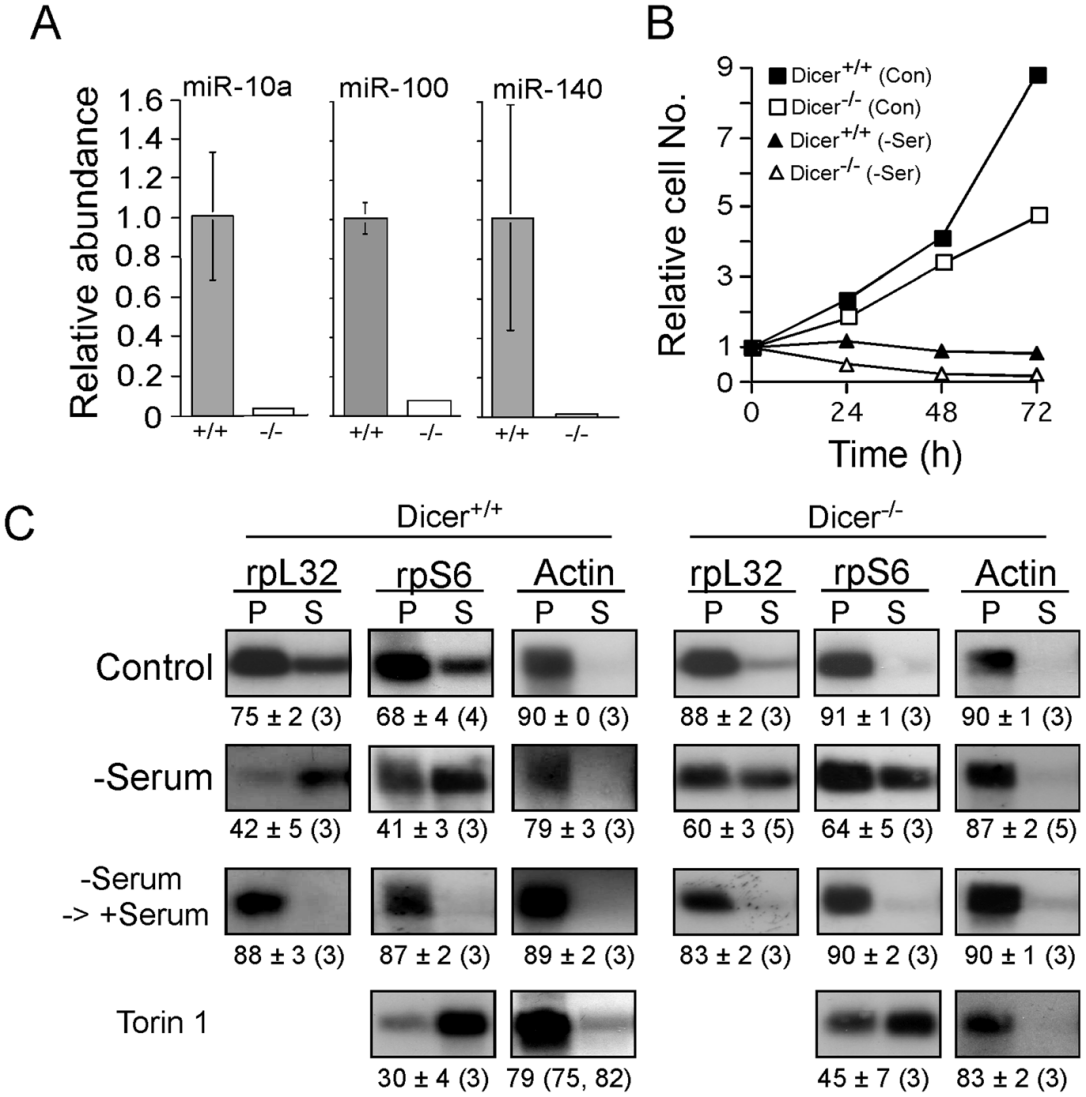
micorRNAs are dispensable for serum-induced translational activation of TOP mRNAs. (A) Total RNA was prepared from Dicer^+/+^ and Dicer^−/−^ MEFs and the relative abundance of the indicated miRs was assessed by quantitative PCR. The abundance of each miR in Dicer^−/−^ MEFs was normalized to that in Dicer^+/+^ MEFs, which was arbitrarily set at one. (B) Dicer^+/+^ and Dicer^−/−^ MEFs were seeded in 96-well plates at a density of 4×10^3^ cells per well. Cells were either untreated (Con) or serum starved (−Ser) for the indicated amount of time. Proliferation was monitored by the methylene blue staining protocol [68]. Absorbance measured 24 h after platting, set arbitrarily at 1, and measured at later time points (average ± SEM [*n* = 5] for each time point) was normalized to that value (note that the error bars are are smaller than the size of the symbols). (C) Dicer^+/+^ and Dicer^−/−^ MEFs were either untreated (Control), serum starved (−Serum) for 48 h or serum starved for 48 h and then serum refed for 3 h (−Serum→+Serum), or treated with 50 nM Torin 1 for 2 h. Cells were harvested and subjected to polysomal analysis using the indicated probes.

The relative inefficient translation of rpL32 mRNA in Drosha knockdown cells (Fig. 7F) could have reflected the requirement for one or more miRs for efficient basal translation of TOP mRNAs, or alternatively, a side effect of the infection by the respective lentivirus. In order to distinguish between these two options, we took advantage of a hemangiosarcoma cell line that had been derived from mouse deficient for Dicer, the cytoplasmic RNase that conducts the second processing step of miRs [55]. These cells, indeed, exhibited an extensive decrease in the abundance of representative miRs (Fig. 8A), yet showed an increase, rather than a decrease, in the basal translation efficiency of both rpL32 and rpS6 mRNAs. These results clearly show that if miRs play a role in this mode of regulation, it is a negative one, rather than a positive one. Consistently, TOP mRNAs were downregulated by serum starvation of Dicer^−/−^ cells to a lesser extent than in their Dicer^+/+^ counterparts and underwent complete recovery following serum refeeding (Fig. 8B). Evidently, the translational repression of TOP mRNAs under serum starvation results from mitotic arrest [56]. However, the relative resistance of Dicer^−/−^ cells to serum starvation cannot be ascribed to an acquired resistance to this stress, as serum starvation had a similar inhibitory effect on the proliferation of both cell genotypes (Fig. 8C). Moreover, it cannot be attributed to the conversion of the mTOR in Dicer^−/−^ cell to a constitutively active one, as it was readily inhibited by Torin 1 (Fig. 8B). Notably, Dicer^−/−^ cells, like iRapKO and iRicKO MEFs, exhibit an inherent resistance to prolonged amino acid starvation (data not shown), and therefore could not be used for a study of the translational behavior of their TOP mRNAs under amino acid deficiency. Collectively, our results with both Drosha knockdown and *Dicer* knockout, indicate that miRs are not required for efficient translation of TOP mRNAs or for their translational activation following stimulation by either amino acid or serum.

## Discussion

Biogenesis of the protein synthesis machinery, and particularly of ribosomes, is a highly resource-consuming process [57]. Thus, cells that encounter unfavorable conditions attenuate the production of components of the translational machinery and cease to grow [58]. Indeed, the present report demonstrates that mTOR-sensitive translational repression of TOP mRNAs is one mechanism that is exploited by cells to selectively downregulate wasteful biogenesis of the protein synthesis machinery under nutritional stresses.

### mTOR operates as a translation activator of TOP mRNA translation outside of its canonical complexes

The positive regulatory role of mTOR in translational activation of TOP mRNAs is supported by results obtained by two complementary experimental approaches: a) the inhibition of amino acid-induced recruitment of TOP mRNAs into polysomes in mTOR knockdown cells or by Torin 1 treatment (Figs. 2D, 4D); and b) the ability of a hyperactive mutant of mTOR to protect the translation of these mRNAs from amino acid deficiency (Fig. 4B). Moreover, the fact that mTOR must be enzymatically active to exert this role indicates that it does not function merely as a scaffold protein (Fig. 4B). However, data presented here demonstrate that the involvement of mTOR in amino acid-activation of TOP mRNA translation depends on neither of the two canonical complexes, mTORC1 and mTORC2 (Figs. 3B). These results coincide with our previous reports on the minor, if at all, reliance of insulin- and oxygen-induced translational activation of TOP on either of these complexes [3], [18]. Not surprisingly, therefore, the role assigned to either S6K or 4E-BP, the two best-characterized mTOR effectors, in translational control of TOP mRNAs has been refuted [59], [60]. A question can be raised as to how amino acid-induced translational activation of TOP mRNA is repressed by rapamycin (Fig. 2) if it does not involve raptor. However, we have already demonstrated that TOP mRNA translation is rendered rapamycin-hypersensitive in raptor-deficient cells [18]. Furthermore, mTOR has been shown to doubly phosphorylate IMP2, another mTOR target, in a raptor-independent fashion. Thus, knockdown of mTOR strongly inhibited IMP2 phosphorylation in cells, whereas depletion of raptor had no effect on this modification *in vitro* or *in vivo* [61]. In light of these supportive data, we propose that mTOR regulates TOP mRNAs translation, and possibly a subset of other targets, through as yet unidentified third complex, or in a complex-independent fashion, and therefore neither raptor nor rictor is critical for its activity toward these targets.

### TSC mediates translational repression of TOP mRNAs under any stress, whereas the RagB transduces only a subset of stimuli to translation efficiency of TOP mRNAs

TOP mRNAs translation, like mTORC1 activity, is protected from oxygen or serum starvation in cells lacking either TSC1 or TSC2 [3], [18]. Contrarily, it has been widely argued that the TSC1-TSC2 complex plays no role in transducing the negative signal resulting from amino acid starvation to mTORC1 activity (reviewed in [62], [63]. However, a recent study has shown that inhibition of mTORC1 by amino acid deprivation is indeed mediated by the TSC1-TSC2 complex. The latter is required for the release of mTORC1 from its site of action, the lysosomal membrane, and therefore, cells lacking TSC fail to efficiently turn off mTORC1 and consequently their response to amino acid starvation is compromised [64]. Results presented here clearly demonstrate that both TSC1 and TSC2 are critical, not only for mTORC1 inhibition, but also for translational repression of TOP mRNAs upon amino acid starvation (Fig. 1). It should be pointed out, however, that since amino acid-induced translational activation of TOP mRNAs does not rely on mTORC1 (Fig. 3), it is conceivable that also TSC1-TSC2 complex exerts its repressive effect on TOP mRNA translation in an mTORC1-indepndent fashion.

Unlike TSC1-TSC2 complex, that convey inhibitory signals to TOP mRNA translation or to mTORC1 activity emanating from all examined stress conditions, RagB^GTP^ can partially derepress TOP mRNAs in cells starved for amino acids or amino acids and serum, but not cells subjected to anoxic conditions (Fig. 6). Evidently, RagB^GTP^ is able to confer on mTORC1 complete resistance to amino acid starvation, and it does so in a raptor-dependent fashion [9]. However, the hierarchical relationships between RagB and mTOR in transduction of amino acids to TOP mRNA translation, a pathway that does not rely on raptor, is yet to be established.

### The nature of the translational *trans*-acting factor of TOP mRNAs is still elusive

The discrete translational behavior of TOP mRNAs implies that the 5′ TOP motif is recognized by a specific translational *trans*-acting factor that transduces physiological cues into a selective translational control of these mRNAs. However, more than two decades of a search for such a regulator(s) have mostly yielded candidates that have failed to withstand the test of time.

The microRNA, miR-10a, was claimed to play both a selective and a positive role in translational control of TOP mRNAs in response to fluctuations in amino acid sufficiency [26]. However, this conclusion has been based primarily on overexpression experiments, which are subject for erroneous conclusions (discussed in [19]). Indeed, data based on loss-of-function approach failed to support the role of miR-10a or its functionally tween, miR-10b, as a determinant of basal translation efficiency of TOP mRNAs (Fig. 7C). Moreover, global microRNA deficiency in Drosha knockdown cells, failed to suppress translational activation of TOP mRNAs upon readdition of either amino acids or serum to starved cells (Fig. 7F). Likewise, experiments conducted with *Dicer* knockout cells revealed that translational activation of TOP mRNAs upon serum refeeding or resuming oxygen supply was not affected by microRNA deficiency (Fig. 8 and [3]). Collectively, these observations appear to disprove a positive role of microRNAs in translational control of TOP mRNAs. Nevertheless, based on the apparent higher translation efficiency of TOP mRNAs in Dicer^−/−^ cells, we cannot exclude the possibility that the basal translation efficiency of these mRNAs is downregulated by one or more microRNAs. Indeed, we have previously shown that Dicer deficiency leads to constitutive activation of mTOR [3], and therefore the higher translation efficiency reported here (Fig. 8B) for Dicer^−/−^ cells might reflect this phenotypic change.

A previous report has demonstrated that two stress granule (SG)-associated RNA-binding proteins, T-cell intracellular antigen-1 (TIA-1) and TIA-1-related (TIAR), assembled onto the first 39 nucleotides of TOP mRNAs in amino acid-starved cells. This interaction resulted in a selective accumulation of TOP mRNAs in SG and their unloading from polysomes, as well as decreased synthesis of TOP mRNA-encoded proteins [65]. Furthermore, simultaneous knockdown of both proteins derepressed TOP mRNA translation in amino acid-, but not oxygen-, deprived cells, implying their critical, yet selective, role in amino acid starved cells [3], [65].

The translational repressor, 4E-BP, is yet another candidate proposed to carry out the translational repression of TOP mRNAs [66]. However, a recently published report, has shown that the TOP mRNAs are translationally regulated by physiologically relevant stresses, like anoxia or serum starvation, in a 4E-BP- independent manner [3].

Finally, LARP1, an RNA-binding protein, has been implicated as positive regulator of TOP mRNA translation [67]. Thus, LARP1 deficiency led to a selective reduced recruitment of TOP mRNAs to polysomes, relative to non-TOP mRNAs, which was accompanied by a decrease in the abundance of TOP mRNA-encoded proteins. However, the selectivity of LARP1 is still questionable, since its silencing reduced the rate of global protein synthesis by 2 to 2.5-fold [67]. Moreover, its activity has not been examined under any physiological stress.

In summary, in light of the conflicting results and the missing information, the nature of the proximal trans-acting factor and its mode of action in translational control of TOP mRNAs still remains elusive.
